# A multi-ingredient containing, proteins, carbohydrate and creatine does not attenuate humoral immune response or performance decrease compared to carbohydrate during resistance training

**DOI:** 10.1186/1550-2783-12-S1-P34

**Published:** 2015-09-21

**Authors:** Nadia Ashrafi, Marcos Seijo, Frank Pullen, Birthe V Nielsen, Joshua Smith, Christian Wilkinson, Yue Fu, Jack Miller, Eneko Larumbe-Zabala, Fernando Naclerio

**Affiliations:** 1 Centre for Sport Science and Human Performance, University of Greenwich, Chatham Maritime, Kent, ME4 4TB, UK; 2 Faculty of Engineering and Science, University of Greenwich, Chatham Maritime, Kent, ME4 4TB, UK; 3 Clinical Research Institute, Texas Tech University Health Science Center, TX, USA

## Background

Nowadays, only carbohydrate has shown to be an effective countermeasure to exercise-induced immune dysfunction while the effect of protein remains controversial. The purpose of this study was to investigate the acute effects of a commercially available multi-nutrient supplement on performance and salivary markers of humoral immunity, following a bout of circuit resistance training in young athletes.

## Methods

Twelve recreationally resistance-trained males (age: 22 ± 1.4 years; body mass 79 ±9.78 kg; 1.81 ± 0.07 m height) volunteered to participate in the study completing 2 randomised controlled circuit resistance training sessions (CT). Participants ingested 2 doses of 500ml of water mixed 60g of a multi-ingredient (MTN) containing whey proteins, carbohydrate, creatine, HMB and sodium bicarbonate or maltodextrin (PL). Beverages were consumed (3 doses of ~166ml) during and after the workout (1 × 500ml). Both MTN and PL looks the same colour and flavour and provide a similar amount of calories (~230 per serving). CT involved three rounds of 7 resistance exercises (CMJs, Bench Press, Parallel-Squat, Upright row, Alternate Lunges, Dead Lift, Push-press, Abdominals) followed by 1 min rest. Participants performed 12 repetitions at 70% 1RM in each of the exercises with no rest in between (only the time to change from one exercise to the next).

Measurements included pre and post (30 min and 60 min) salivary markers of humoral immune response: Antimicrobial Peptide, Alpha Defensins (HNP 1-3). The total kg lifted per exercise and in the overall workout was considered as indicator of performance. ANOVA design and Cohen d effect sizes (ES) were used to analyse potential differences between times and treatment conditions.

## Results

No significant differences were observed between the total weight (kg) lifted per exercise or for the entire session (p > 0.05). HNP 1-3 showed a strong trend (p = 0.06) with a moderate effect size (d = 0.53) at 30 min for the CHO condition [2.001 (1.95) vs 3.037 (2.49) ng/mL], nevertheless, no significant differences were observed at 60 min with respect to the values measured at both pre [3.825 (3.21) vs 2.001 (1.95) ng/mL] and 30 min [3.825 (3.21) vs 3.037 (2.49) ng/mL]. On the other side, HNP 1-3 did not increase at either 30 min [2.464 (3.31) vs 3.656 (3.22) ng/mL] or 60 min [2.464(3.31) vs 2.387 (2.46) ng/mL] post workout for the MTN treatment condition. No differences were observed between the two tested treatment conditions for the three analysed times points (pre, post 30 min and post 60 min).

## Conclusion

Ingesting both MTN and CHO supplements during and after a circuit resistance-training workout, resulted in no impact on performance. However, even when both nutritional interventions were effective to attenuate the increase of antimicrobial peptide alpha-defensins, MTN showed a stronger effect to blunt exercise-induced immune-dysfunction. These results did not support the notion that only carbohydrate with no added proteins is the only effective nutritional countermeasure against the transient post exercise immunosuppression.

